# Neurotrophic and Neuroprotective Effects of *Hericium erinaceus*

**DOI:** 10.3390/ijms242115960

**Published:** 2023-11-03

**Authors:** Izabela Szućko-Kociuba, Alicja Trzeciak-Ryczek, Patrycja Kupnicka, Dariusz Chlubek

**Affiliations:** 1 Institute of Biology, University of Szczecin, 13 Wąska, 71-415 Szczecin, Poland; alicja.trzeciak-ryczek@usz.edu.pl; 2 The Centre for Molecular Biology and Biotechnology, University of Szczecin, 13 Wąska, 71-415 Szczecin, Poland; 3 Department of Biochemistry and Medical Chemistry, Pomeranian Medical University in Szczecin, Powstańców Wlkp. 72, 70-111 Szczecin, Poland; patrycja.kupnicka@pum.edu.pl (P.K.); dariusz.chlubek@pum.edu.pl (D.C.)

**Keywords:** lion’s mane mushroom, nerve system, neurotrophins, secondary metabolites

## Abstract

*Hericium erinaceus* is a valuable mushroom known for its strong bioactive properties. It shows promising potential as an excellent neuroprotective agent, capable of stimulating nerve growth factor release, regulating inflammatory processes, reducing oxidative stress, and safeguarding nerve cells from apoptosis. The active compounds in the mushroom, such as erinacines and hericenones, have been the subject of research, providing evidence of their neuroprotective effects. Further research and standardization processes for dietary supplements focused on *H. erinaceus* are essential to ensuring effectiveness and safety in protecting the nervous system. Advancements in isolation and characterization techniques, along with improved access to pure analytical standards, will play a critical role in achieving standardized, high-quality dietary supplements based on *H. erinaceus*. The aim of this study is to analyze the protective and nourishing effects of *H. erinaceus* on the nervous system and present the most up-to-date research findings related to this topic.

## 1. Introduction

Neuroprotective action refers to the ability of substances or factors to shield neurons from damage or death. The goal is to prevent the degeneration of nerve cells and maintain proper neural functioning. Neuroprotection can be achieved through a variety of mechanisms, such as reducing oxidative stress, inhibiting inflammation, regulating apoptosis, and improving mitochondrial function and blood flow to the brain. One such aspect is neurotrophic action, which refers to the ability of substances or factors to stimulate the growth, differentiation, and function of neurons. The balance between neurodegenerative and neuroregenerative processes largely depends on the availability and activity of neurotrophic factors that are essential for maintaining the functional organization of neurons. By interacting with receptors on nerve cells and influencing their survival, synaptic functions, and neuronal plasticity, these substances play a crucial role in the development, maintenance, and regeneration of the nervous system. Neurotrophic factors such as nerve growth factor (NGF), brain-derived neurotrophic factor (BDNF), and glial cell-derived neurotrophic factor (GDNF) can support neuron growth and development as well as protect neurons from damage and degeneration. Therefore, substances that are similar to neurotrophic factors or their inducers can be utilized in the treatment of neurodegenerative diseases [1,2].

Neurodegenerative diseases encompass a broad group of disorders leading to progressive loss of structure and function of nerve cells in various regions of the brain and/or spinal cord, such as Parkinson’s disease (PD), Alzheimer’s disease (AD), Huntington’s disease, amyotrophic lateral sclerosis, multiple sclerosis, and Creutzfeldt-Jakob disease. Each of these manifests with characteristic symptoms and pathologies, but they all share a common factor—the gradual loss of nerve cells. These serious and often debilitating conditions affect millions of people worldwide, impacting not only the patients but also their families and caregivers. Associated with the degeneration and death of neurons, neurodegenerative diseases lead to impaired neural communication and brain function and are characterized by a gradual decline in cognitive, motor, and/or autonomic functions. Their courses vary but are often associated with a progressive deterioration of health and loss of independence. The causes of such diseases are complex and multifactorial, involving genetic predispositions, environmental factors, aging processes, and abnormal accumulation of proteins or other pathological changes in the brain. While there is currently no known way to completely cure neurodegenerative diseases, available therapies often aim to alleviate symptoms, delay disease progression, and improve patient quality of life. Ongoing research aims to understand the mechanisms of disease development and develop new therapies. The quest for effective methods of treatment and prevention is a significant area of scientific and clinical research associated with the immense impact of these conditions on public health and the quality of life of many people worldwide.

The use of mushrooms in neuroprotection is a subject of intensive scientific research aiming to identify potential health benefits and understand the mechanisms of their action at the neuronal level. Studies indicate that some mushroom species demonstrate promising neuroprotective properties, opening possibilities for their use in the prevention and treatment of neurological disorders [3,4].

Mushrooms have been utilized in traditional medicine for centuries. *Hericium erinaceus* (Bull.: Fr.) Pers., also known as lion’s mane mushroom, bearded tooth mushroom, “Yamabushitake” in Japan, and “Houtou” in China, is a medicinal and edible mushroom belonging to the Basidiomycota phylum within the *Hericiaceae* family. It is a saprotroph, but it can occasionally act as a mild parasite on trees, e.g., on dead or dying trees of the genera *Quercus* sp., *Fagus* sp., *Acer* sp., *Juglans* sp., and *Ulmus* sp. [5]. It occurs naturally in Asia, Europe, and North America, and in 2003 it was included in the red list of endangered and extinct species in 13 out of 23 European countries due to the decrease in its natural habitats [6].

*H. erinaceus* can be cultivated on a large scale using inexpensive substrates, such as artificial and agricultural waste. Commercial cultivation of this species is popular worldwide, especially in Asia and the USA [7]. Due to its beneficial properties, this mushroom is widely used in the diets of East Asian countries [8,9]. In traditional Chinese and Japanese medicine, lion’s mane mushroom has been used for centuries as a remedy for gastrointestinal disorders, liver and kidney diseases, spleen disorders, and heart regulation. Indigenous Americans in North America also used it as a preventive measure against bleeding. 

In the last decade, there has been a growing interest in the potential use of *H. erinaceus* in the therapy of various neurological and cognitive disorders. Moreover, it is suggested that they may have a neuroprotective action, which refers to the ability of substances or factors to protect and support the health of nerve cells and prevent their damage. Research shows that *H*. *erinaceus* exhibits numerous therapeutic properties, such as antioxidative [10], anti-inflammatory [11], hypolipidemic [12], hemagglutinating [13], antimicrobial [14], hypoglycemic and antidiabetic [15,16,17], and anticancer effects [18,19,20]. Additionally, H. erinaceus induces the synthesis of nerve growth factor (NGF), inhibits β-amyloid (Aβ) cytotoxicity, and protects nerve cells from death caused by oxidative stress or endoplasmic reticulum stress. 

**Figure 1:**
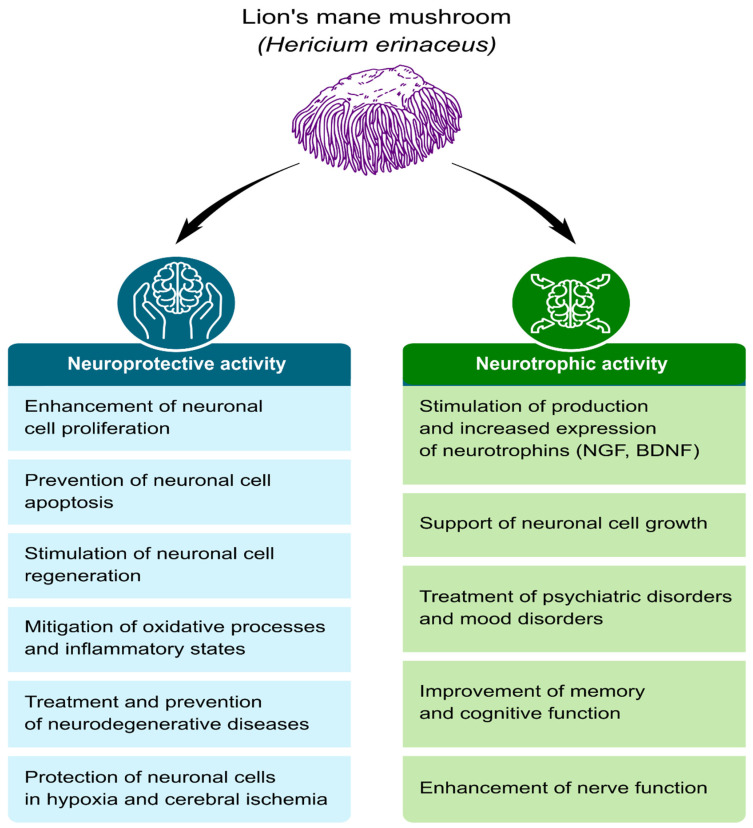
Examples of the neuroprotective and neurotrophic effects of the Lion’s Mane mushroom in preclinical studies [2,15,26,30,31,32,33,34,35,36,37,38,39,40,41,42,43,44,45,46,47,48,49,50,51,52,53,54,55,56,57,58,59,60,61].

Positive effects of *H*. *erinaceus* have been observed in the treatment of cognitive disorders, Alzheimer’s disease, ischemic strokes, Parkinson’s disease, and age-related hearing impairment [15,21,22,23,24]. Promising results have also been obtained in the treatment of depressive disorders [25,26,27,28,29]. The studies referred to above were conducted on animal models (Figure 1).

Given the abundance of research dedicated to *H. erinaceus*, it is necessary to provide a current summary of the latest and most significant research findings regarding this organism and its impact on the vertebrate nervous system.

## 2. Neurotrophins

Neurotrophins play a significant role in the survival of specific neuron populations and promote the growth and branching of their axons (also known as neurites), which play a crucial role in the development and proper functioning of the nervous system, such as the communication between neurons. Neurotrophins are synthesized in both the central and peripheral nervous systems and are essential for dendritic branching, synaptogenesis, synaptic plasticity, and cellular homeostasis control. In general, this family of proteins plays a key role in neurogenesis processes such as neuronal differentiation, maturation, and survival [62,63]. They act at various stages of neurogenesis, and their concentration can be regulated by other modulating factors [35]. In mammals, within the family of neurotrophins, we can distinguish NGF, BDNF, neurotrophin 3 (NT3), and neurotrophin 4/5 (NT4/5) [29,64].

### 2.1. NGF and BDNF

Nerve growth factor (NGF) is a neurotrophic factor that regulates the differentiation, growth, survival, and plasticity of cells such as cholinergic neurons in the septum, striatum, and nucleus basalis of Meynert [65]. The highest levels of NGF-mRNA synthesis and NGF protein have been observed in the brain, particularly in the basal forebrain, hippocampus, cerebral cortex, and olfactory bulb [63,66]. 

NGF is secreted tonically as a monomer in its precursor form (proNGF) and undergoes extracellular maturation by plasmin to become the active form (mNGF). Nerve growth factors are unable to cross the blood-brain barrier and are easily metabolized by peptidases. Consequently, their use as drugs in the treatment of neurodegenerative diseases may be challenging. For this, research is being conducted on compounds that promote NGF synthesis [67]. 

Currently, it is believed that dysfunctions in extracellular NGF metabolism may lead to accelerated degradation of mature NGF in Alzheimer’s disease. Cholinergic neurons require NGF for proper functioning and plasticity, and NGF metabolism is disrupted in Alzheimer’s disease. Changes in neurotrophin-related signaling pathways are involved in the aging process and contribute to the cognitive decline observed in Alzheimer’s disease. Functional NGF deficiency is believed to be associated with Alzheimer’s disease and plays a role in the pathogenesis of this condition. Studies have evidenced reduced levels of NGF in the basal forebrain in patients with Alzheimer’s disease. Additionally, in patients without dementia symptoms but with amyloid plaques, reduced NGF levels were observed in the frontal cortex [36,65].

NGF has been shown to play a role in maintaining long-term synaptic potentiation (LTP) in the dentate gyrus. NGF blockade in the hippocampus leads to a decrease in LTP and impairment of spatial memory [68]. Additionally, studies have shown that exogenous infusion of NGF into the hippocampus enhances memory, and increased NGF expression correlates with the learning process [69].

BDNF and NGF are the two best-known neurotrophins. In mammals, the highest expression level of BDNF protein is found in the brain, particularly in the hippocampus and cerebral cortex, which are responsible for memory, learning, and higher cognitive processes. Other peripheral sources of BDNF include the lungs, heart, spleen, liver, thymus, gastrointestinal tract, skin, and smooth muscle tissue in blood vessels [70]. 

BDNF is synthesized as a precursor protein, pro-BDNF, which is converted by proteases into its mature form [37]. BDNF, along with its receptor TrkB, plays a significant role in long-term synaptic potentiation (LTP), which is considered a model process in memory formation [29]. Additionally, BDNF plays an essential role in mood regulation, which closely correlates with the neurogenic actions of antidepressant drugs. Dysfunctions in the BDNF pathway have been linked to Alzheimer’s disease, schizophrenia, Huntington’s disease, and Rett syndrome [29].

### 2.2. The Mechanisms of NGF and BDNF Activity

The trophic activity of both NGF and BDNF is mediated by interactions with Trk receptors (transmembrane proteins with tyrosine kinase activity) and p75 receptors (a transmembrane glycoprotein that acts as a low-affinity receptor for NGF) [38,71,72]. NGF activates the TrkA receptor, while BDNF activates TrkB [73]. Both neurotrophins also activate the p75 receptor with low affinity, which is located on the plasma membrane of axonal terminals (presynaptic membrane) and is co-expressed with Trk receptors, allowing them to promote enhanced trophic signaling, resulting in increased neuronal survival, neurite growth, and synaptogenesis [29]. The p75NTR receptor lacks a catalytic domain for autoactivation, so its functions depend on interactions with other receptors [74]. 

Activation of either receptor by NGF leads to different outcomes. The trophic signal that induces cell differentiation and survival is conveyed through the TrkA receptor. It is also believed that the role in learning and memory processes is mediated through the activation of the high-affinity TrkA receptor. Stimulation of only p75NTR receptors can elicit trophic effects, such as promoting neuronal migration and differentiation, but similarly to the stimulation of other TNF family cytokine receptors, it can also lead to sphingolipid hydrolysis, ceramide production, and ultimately, programmed cell death [69,73]. 

Pro-BDNF exerts its actions through binding to the p75 neurotrophin receptor, while BDNF acts through TrkB receptors, which are widely expressed in various parts of the brain [37]. BDNF, by binding to TrkB, triggers the activation of the Ras/Raf/MAKP, PLCg, and PI3K/Akt signalling cascades, which play roles in neurogenesis, neuronal survival, and plasticity. Reduced BDNF expression has been demonstrated in patients with Alzheimer’s disease in the hippocampus, dentate gyrus, new cortex, and nucleus basalis of Meynert [39]. Defects in the mechanism of pro-BDNF conversion to BDNF, or imbalances between these two forms, have been correlated with impaired cognitive function, psychiatric disorders, and anxiety symptoms [37].

Given the above, neurotrophins, along with their receptors, are considered promising therapeutic targets for the treatment of neurological and neurodegenerative diseases. While exogenous therapies based on neurotrophins have not yielded the expected results in clinical trials, mainly due to the short half-life of neurotrophins and low permeability of the blood-brain barrier (BBB) [29], scientists are exploring alternative interventions based on plants and fungi that can increase endogenous BDNF levels and TrkB receptor activity. 

A promising approach may involve the use of natural compounds with potential neuroprotective properties. Nutraceuticals that exhibit multidirectional biological effects may contribute to improved cognitive function. Such substances are safe, readily available, and relatively inexpensive compared to bioengineering methods, making them potentially effective therapeutic agents for maintaining brain health. The discovery of neurotrophin-stimulating compounds, such as hericenones isolated from the fruiting bodies of *H. erinaceus* and erinacines from mycelia, has made this mushroom species valuable for research purposes.

## 3. Activities of Components Isolated from *Hericium erinaceus*

*H. erinaceus* is characterized by its high nutritional value. According to a study conducted by Friedman [30], dried fruiting bodies of this mushroom contain approximately 61.1% carbohydrates, 5.1% fatty acids, 20.8% proteins, 6.2% water, and 6.8% ash on a dry weight basis, providing around 374 kcal/100 g. On the other hand, dried mycelium contains about 42.5% proteins, 42.9% carbohydrates, 6.3% fatty acids, 3.9% water, and 4.4% ash, providing around 398 kcal/100 g. In terms of carbohydrates, it is worth noting that lion’s mane mushroom exhibits a high content of arabitol, up to 127 mg/g dry weight, as shown in studies by Mau et al. [75] and Valu et al. [76]. Additionally, detailed research conducted by Valu et al. [76] identified the presence of 19 amino acids and 32 aromatic substances in lion’s mane mushrooms. Both the fruiting bodies and the mycelium of *H. erinaceus* contain a range of macro- and microelements. According to Eisenhut et al. [77], lion’s mane mushroom contains significant amounts of potassium (254 mg/100 g dry weight) and phosphorus (109 mg/100 g dry weight), while the content of magnesium, zinc, and copper is lower. Furthermore, cobalt, iron, manganese, molybdenum, selenium, sodium, and sulfur have also been detected in lion’s mane mushrooms. It is worth noting that lion’s mane mushrooms may also contain heavy metals such as arsenic, cadmium, copper, and lead. As suggested by Yang et al. [78], the content of heavy metals in mycelium is higher than in the fruiting bodies.

Studies on the content of secondary metabolites in *H. erinaceus* have been conducted since the 1990s. In 2021, Yang et al. [79] identified as many as 102 compounds present in this mushroom. Among these compounds were organic acids, nucleotides and their analogs, amino acids, carbohydrates and their derivatives, flavonoids, unsaturated fatty acids, terpenoids, phenolic acids, phenylpropanoids, and steroids. 

Research has shown the presence of a range of bioactive substances in both the fruiting bodies and mycelium of *H. erinaceus* (Table 1). These substances can be divided into two main categories. The first category consists of high-molecular-weight compounds, such as polysaccharides such as β-glucans, as well as other polysaccharides and polypeptides, which have a significant impact on strengthening the body’s immune system. The second category consists of low-molecular-weight compounds, such as terpenoids and polyketides, including erinacines and hericenones, which exhibit antioxidative, antidiabetic, anticancer, anti-inflammatory, and hypolipidemic properties, as shown by studies conducted by Thongbai et al. [6] and Ratto et al. [31]. These compounds have the ability to interact at the molecular level by regulating cytokines, protein kinases, and transcription factors. It is particularly interesting that the most abundant compounds, hericenones and erinacines, are capable of effectively crossing the blood-brain barrier (BBB) [80]. They demonstrate neuroprotective and neurotrophic effects, both in vitro and in vivo, in animal models of peripheral nerve injury [32], stroke [33], and Alzheimer’s disease [15,30,34]. *H. erinaceus* may exhibit pharmacological activity at the tissue, organ, and systemic levels, as suggested by the results of research conducted by Roda et al. [81] (Figure 1).

### 3.1. Polysaccharides 

Polysaccharides in fungi are mainly present in the cell wall and can constitute up to 20% of the mass in both fruiting bodies and mycelium [6]. Polysaccharides with antitumor properties have been isolated from the basidia of *H. erinaceus*, such as xylans, glucans, heteroxyloglucans, and galactoxyloglucans [6,88].

In 2004, Jia et al. [89] isolated heteropolysaccharides with a mass of 18 kDa, such as rhamnose, galactose, and glucose, while Zhang [90,91] found some with a mass of 19 kDa containing fructose, galactose, and glucose. In *H. erinaceus* from Malaysia, arabinoxylans are the main sugar component, with glucose, rhamnose, deoxyribose, and galactose being much less common [92]. Polysaccharides isolated from *H. erinaceus* served various functions, such as neurogenesis, peripheral nerve regeneration, and muscle regeneration after injuries [50], as well as immunostimulatory, anticancer, and cholesterol-lowering activities [93] Hou et al. [94] isolated water-soluble oligosaccharides from *H. erinaceus* with antioxidant properties.

### 3.2. Erinacines

Erinacines are a group of bioactive compounds capable of crossing the blood-brain barrier and inducing the expression of NGF in the brain [95]. These compounds are obtained from the mycelium and fruiting bodies of *H. erinaceus* and belong to the group of substances known as cyathane-xyloside, which are diterpenoids. These molecules contain five-, six-, and seven-membered rings in their structure, with cyathane-xyloside additionally containing xylose (wood sugar) attached to the aglycone [52].

**Figure 2:**
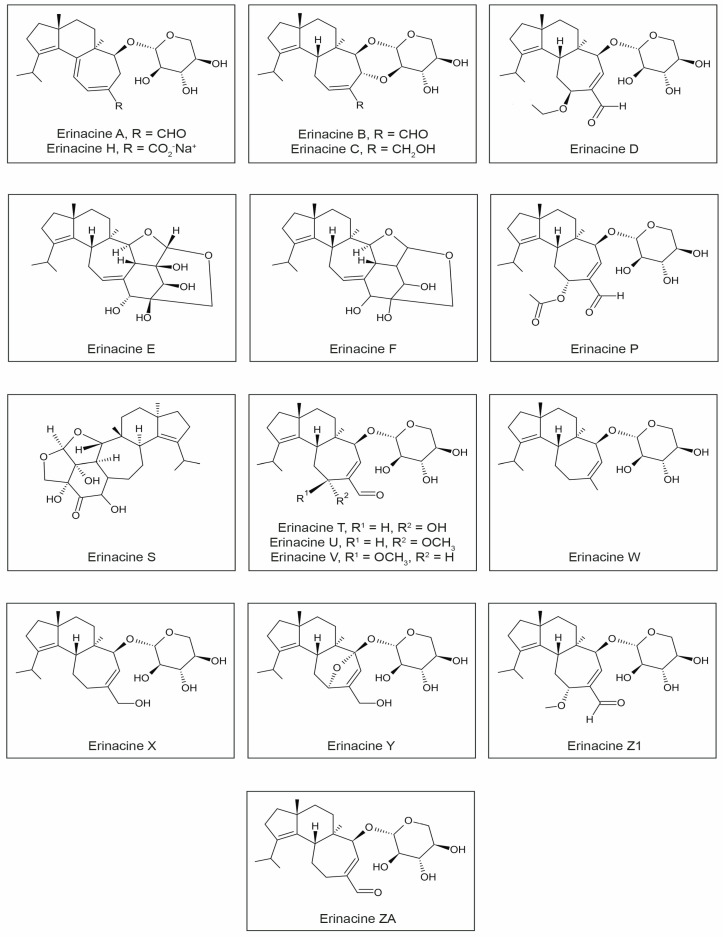
Chemical structures of erinacines.

Three main biological activities of erinacines have been identified. Firstly, they demonstrate the ability to stimulate NGF synthesis. Secondly, they possess antibiotic properties. Thirdly, they can stimulate the κ opioid receptor. So far, nineteen different erinacines have been isolated. Among these compounds, ten exhibit neuroprotective activity, including the stimulation of NGF synthesis (erinacines A, B, C, E, F, and H) and the promotion of neurite outgrowth (T, U, V, and P) (Figure 2, Table 1).

Erinacine A, a natural NGF synthesis inducer with low molecular weight, showed effectiveness in several mouse models of age-related neurological diseases using oral administration [20,22,96]. Some erinacines (A and S) also exhibit actions that reduce β-amyloid deposition and increase the expression of the gene encoding the insulin-degrading enzyme. Erinacine A is exclusively present in the fermented mycelium and is absent in *H. erinaceus* fruiting bodies. Erinacine E has found application in the treatment of neuropathic pain in animal models [35,40,45,46,97,98,99,100,101]. Kenmoku et al. [100,101] demonstrated that in the mycelium of *H. erinaceus*, erinacine Q serves as a direct substrate for the biosynthesis of erinacine P, which is a parent precursor for other important erinacines and striatins (striatal derivatives). In a study by Ma et al. [47], erinacines W, X, Y, and ZA showed significant neurotrophic effects on PC12 cells (Figure 2). 

### 3.3. Ergothioneine

The fruiting bodies and mycelium of *H. erinaceus* in the presence of ergothioneine, an organic compound belonging to the group of amino acids and betaine, have been observed. It exhibits antioxidant and cytoprotective properties. A growing number of scientific reports suggest the potential application of therapy based on L-ergothioneine in the treatment of cardiovascular diseases [102], musculoskeletal disorders, pre-eclampsia [103], and neurodegenerative diseases [81,104,105].

### 3.4. Hericenones

**Figure 3:**
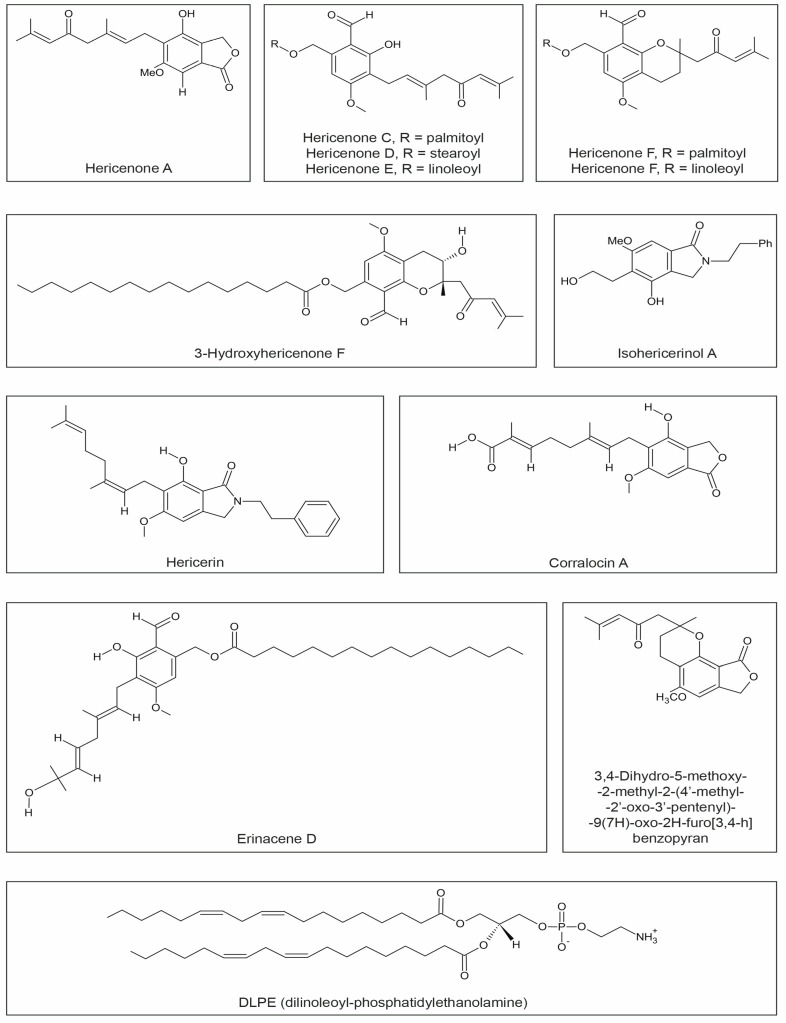
Chemical structures of hericenones, 3-hydroxyhericenone F, isohericerinol A, hericerin, corralocin A, erinacene D, 3,4-Dihydro-5-methoxy-2-methyl-2-(40-methyl-20-oxo-30-pentyl)-9(7H)-oxo-2h-furo[3,4-h]benzopyran, and DLPE.

From the fruiting bodies of *H. erinaceus*, several hericenones (A, B, C, D, E, F, G, H, I, J) (Figure 3) have been isolated, which are phenolic derivatives with diverse biological activities. *H. erinaceus* is the sole source of these valuable acids [40,83,84,106]. Hericenones A and B exhibit cytotoxic properties against HeLa cervical cancer cells. Hericenones C-E and H stimulate nerve growth factor (NGF) synthesis and NGF gene expression through the activation of the protein kinase A signaling pathway. The activity of individual hericenones differs depending on the length and presence of double bonds in the fatty acid chains. The highest NGF-stimulating ability was observed in hericenones E, which possess two double bonds in the chain [35].

Approximately 3-hydroxyhericenone F (Figure 3) demonstrates protective activity on cells against endoplasmic reticulum (ER) stress-induced apoptosis, which is implicated in neuronal apoptosis in many neurodegenerative diseases, including Alzheimer’s, Parkinson’s, Huntington’s, and prion diseases [84]. Moreover, other phenolic compounds, such as hericenones A-D, hericenals A and B, hericenol A, erinacerines A and B, and hericerin, have been isolated from *H. erinaceus*. Hericenes B and C exhibited neuroprotective properties against ER stress induced by tunicamycin [106,107] (Figure 3, Table 1). 

### 3.5. Dilinoleyl-Phosphatidylethanolamine (DLPE)

DLPE (Figure 2), isolated from the fruiting bodies of *H. erinaceus*, is a phospholipid containing two unsaturated fatty acids, namely linoleic acids. It exhibits properties that protect neurons from cell death caused by ER stress [15], wherein a protective mechanism involving the PKC (protein kinase C) pathway is engaged. There is a connection between this protective mechanism and the activation of protein kinase C (PKC) by phospholipids, mainly phosphatidylserine and unsaturated fatty acids [38,85]. Some researchers suggest that PKC reduces cell mortality by reducing the toxicity of amyloid beta [108,109], while others believe that PKC contributes to apoptosis induction [110].

### 3.6. Other Compounds

Li et al. [111] isolated new aromatic compounds from *H. erinaceus*, such as erinacene D (Figure 2), and demonstrated that this compound has the ability to inhibit the transcriptional activity of NF-kB induced by tumor necrosis factor-alpha (TNFα), confirming their potential role in NF-kB activity inhibition (Table 1). 

Steroids such as six erinarols (A-F), five ergostane-type sterol fatty acid esters, ten ergostane-type sterols, and four erinarols (G-J) have also been isolated from the fruiting bodies of *H. erinaceus*. All these compounds exhibit anti-inflammatory and antiproliferative properties [112]. 

Furthermore, nucleoside components have been isolated, showing diverse forms of biological activity, such as anticancer and antiviral effects, as well as potential use in gene therapy. Adenosine, inosine, and guanosine exhibit a variety of pharmacological effects, including immune regulation, neuroprotection, and potential treatment for cardiovascular diseases. Among the compounds isolated from *H. erinaceus*, flavonoids have also been identified, which can be utilized in pharmacology, including for neuroprotection, treatment of myocardial ischemia, improvement of cognitive function, prevention of chronic gastric ulcers, protection of reproductive tissue, as well as acting as anti-inflammatory and anticancer drugs [79].

## 4. The Neuroprotective and Neurotrophic Potential of *H. erinaceus* Components

The impact of extracts derived from *H*. *erinaceus* has been extensively investigated for many years, both in in vitro and in vivo studies as well as clinical research. This subsection summarizes the physiological mechanisms and behavioral effects induced by the action of various types of extracts obtained from *H*. *erinaceus* (Table 2).

### 4.1. Growth, Regeneration, and Protection of Nerve Cells

Since the 1990s, several in vitro studies have been conducted, demonstrating the stimulatory effects on the synthesis of nerve growth factor (NGF) by hericenones (C, D, E, H) and erinacines (A-F, H) extracted from *H. erinaceus* (Table 1) [40,43,44,45,48]. However, these studies did not provide a clear understanding of the underlying mechanisms of action or how they influence neurite growth. Rupcic et al. [41] analyzed the effects of secondary metabolites from *H. erinaceus* on 1321N1 cells and confirmed previous findings suggesting that erinacines A, B, C, and E stimulate NGF synthesis. Moreover, they were the first to demonstrate that erinacine Z1 also increases the expression of this neurotrophin. Additionally, they found that erinacine C also increases the expression of BDNF. The ability of erinacine to stimulate the transcription of both neurotrophins suggests the existence of a common regulatory factor that plays a role in inducing both NGF and BDNF. Similar neurotrophin expression stimulation was observed by Ryu et al. [86]. They isolated hericerin, isohericerinol A, and corallocin A from *H. erinaceus*. Isohericerinol A and corallocin A increased the expression of BDNF, while hericerin and isohericerinol A increased the level of NGF.

Rat pheochromocytoma (PC12) cells are often used as an in vitro model in research on neurodegenerative diseases because they have the ability to form synapses and produce proteins related to the nervous system [9,60]. Phan et al. (2014) [35] conducted a study to examine neurite growth activity and analyze signaling pathways involved in neurogenesis after *H. erinaceus* induction in PC12 cells. They found that hericenone E exhibits neurotrophic effects in these cells. This action is associated with the stimulation of nerve growth factor (NGF) synthesis and subsequently increased phosphorylation of the TrkA receptor by NGF, leading to the activation of ERK and Akt signaling pathways. Additionally, they analyzed additional signaling events and found that hericenone E activates the ERK1/2 and PI3K/Akt cascades independently of the presence of NGF.

The stimulation of ERK activity by hericenone E independently of NGF suggests that this compound is involved in additional signaling pathways directly regulated by hericenone E. These signaling pathways complement the ERK and Akt pathways, leading to neuronal differentiation and neurite growth. Zhang et al. [46] demonstrated the significant effects of erinacines T, U, V, and P on neurite growth in PC12 cells. Furthermore, Ma et al. [47], using genetic engineering techniques, synthesized new types of erinacines (W, X, Y, and ZA) in *Saccharomyces cerevisiae*, which also exhibited neurotrophic effects on PC12 cells. Studies on the effect of *H. erinaceus* on neurite elongation have been conducted by Rahman et al. [10], Zhang et al. [14], and Lai et al. [2], and all of them confirmed this effect (Table 2).

Cheng et al. [59] investigated the effects of different concentrations of *H. erinaceus* polysaccharide extracts (HEPS) and Aβ1-40 on PC12 cells to induce cytotoxicity. The accumulation of Aβ plays a significant role in initiating and developing Alzheimer’s disease (AD). Neurotoxic mechanisms associated with this disease include oxidative stress and mitochondrial dysfunction, leading to apoptosis and neuronal dysfunction. The researchers found that HEPS promoted the survival of PC12 cells under toxic conditions induced by Aβ. Moreover, they observed increased effectiveness in removing free radicals and reactive oxygen species (ROS). As a result, HEPS protected PC12 cells from Aβ-induced apoptosis. In vivo experiments were also conducted on APPswe/PS1dE9 mice to assess the impact of erinacine A on Alzheimer’s disease. They showed that after 30 days of oral administration of *H. erinaceus* mycelium, the insulin-degrading enzyme (IDE) expression was enhanced, and the amount of Aβ amyloid plaques in the brain was reduced. Additionally, an increased ratio of nerve growth factor (NGF) to its precursor pro-NGF and an increase in the number of new neurons in the dentate gyrus (DG) were observed. Furthermore, after 81 days of *H. erinaceus* supplementation, improvements were seen in the impaired brain regions of transgenic mice, resulting in the reversal of behavioral deficits [22].

The neuroprotective and therapeutic efficacy of erinacine A in improving pathological conditions and behavioral deficits in Parkinson’s disease (PD) and Alzheimer’s disease (AD) was also confirmed in an animal model (C57BL/6 mice) by Lee et al. [23]. The researchers conducted studies on the potential use of erinacine A in the treatment of Parkinson’s disease. In vitro experiments were performed on N2a cells, and in vivo experiments were performed on a C57BL/6 mouse model. These studies were the first to demonstrate that erinacine A can reduce MPTP-induced neurotoxicity by activating cell survival pathways such as PAK1, AKT, LIMK2, and MEK and by reducing cell death pathways such as IRE1α, TRAF2, ASK1, GADD45, and p21. Li et al. [96] developed an in vitro model to confirm the effectiveness of EAHE and demonstrated that, in the absence of NGF, EAHE extract from the mycelium was capable of inducing neurite outgrowth in primary cultures of rat cortical neurons in a concentration-dependent manner (Table 2).

Chen et al. [52] and Tzeng et al. [133] conducted animal studies that showed the neuroprotective effects of erinacine S in neurodegenerative diseases. Further, Lin et al. [82] performed in vitro experiments on primary neurons from mice and rats. This study demonstrated that after incubation with erinacine S, there was a significant increase in neurite outgrowth in both central nervous system (CNS) and peripheral nervous system (PNS) neurons. They also analyzed the mechanism of this phenomenon using the RNA-seq technique and confirmed through ELISA that erinacine S stimulates the accumulation of neurosteroids. It was also shown that neurosteroids stimulate neurite outgrowth, induce neurogenesis, and prevent neuronal apoptosis, which explains the neuroprotective effect of erinacine S in Alzheimer’s disease and neuronal regeneration. Additionally, it was suggested that erinacine E exhibits strong and selective agonistic activity on κ-opioid receptors present in the peripheral endings of major ascending neurons. This activity indicates the potential use of erinacine E as an analgesic substance. Moreover, it was suggested that κ-receptor agonists may have the potential to act as neuroprotective agents in conditions such as brain hypoxia and ischemia (Table 2) [43,44].

Park et al. [58] analyzed the neurotrophic activity of compounds derived from *H. erinaceus* fruiting bodies in rat hippocampal cells. It was shown that the addition of the polymer could partially delay the apoptosis of nerve cells and lead to an increase in the number of cells containing neurites, ultimately contributing to the growth of nerve cells. Studies were conducted on the neurotrophic effects of dried *H. erinaceus* fruiting bodies on neurons in rat hippocampal slices by analyzing cellular impulse activity. The results demonstrated that the fruiting bodies exhibited neurotrophic or stimulating effects on neurons at concentrations that had no effect on the growth of nerve cells in vitro and did not induce toxic effects or damage to nerve cells. Neurons in the hippocampus, which are part of the limbic system, play a significant role in the regulation of motivational-emotional responses, memory, and other cognitive functions [32,134]. A characteristic feature of this structure is its remarkable sensitivity to even minor changes in the composition of the intercellular fluid substance, which is much greater than in the case of neurons in the cerebral cortex and cerebellum. The extract also promoted the normal development of cultured cerebellar cells and showed a regulatory effect on myelination processes in vitro after myelin damage. The myelin sheath plays an important role in transmitting nerve signals. Damage to the compact myelin structure leads to impairment and serious nervous system diseases [116].

Shimbo et al. [123] tested the effect of erinacine A on different brain regions in rats and observed an increase in NGF synthesis in certain regions. This could be attributed to two factors: the stimulating effect of erinacine A on noradrenaline synthesis, leading to increased NGF secretion in the hippocampus, and the locus coeruleus (LC) region. Erinacine A may act at the neurotransmitter-neurotrophin level, as evidenced by studies showing that adrenergic receptors have been identified in astrocytes, and noradrenaline (NA) regulates neurotrophin synthesis in the whole brain and in astrocytes in the hippocampus. Given that adrenergic receptors are present in hippocampal neurons (associated with the hippocampus), it is possible that NA regulates neurotrophin synthesis in hippocampal glial and nerve cells. Another mechanism suggests that erinacine A increases the levels of neurotrophin 3 (NT-3) in the LC as well as enhances the survival of noradrenergic neurons and NA synthesis in the LC. Noradrenaline in the LC stimulates NGF synthesis in the hippocampus.

Neurotrophin-3, in turn, induces the formation of new receptors for the tyrosine kinase C enzyme, recognizing other neurotrophic factors, such as NGF, and simultaneously activating them. A study conducted by Mori et al. [36] showed that oral administration of *H. erinaceus* to mice for 7 days increased the expression of genes encoding NGF fivefold in the hippocampus. Interestingly, this effect was observed only in the hippocampus, not in the cerebral cortex, which suggests a significant influence of NGF on memory and learning, especially considering that in mammals, neurogenesis occurs in the hippocampus, specifically in the dentate gyrus area [135]. Furthermore, *H. erinaceus* extracts have shown the ability to induce phosphorylation of c-Jun N-terminal kinase (JNK) and its substrate, c-Jun protein, as well as increase c-Fos expression. These results suggest that *H. erinaceus* stimulates the expression of the NGF gene through JNK signaling (Table 2) [36].

Wong et al. [55] conducted a study on the effects of *H. erinaceus* extracts on the NG108-15 cell line, characterized by high proliferative activity and rapid neurite growth. In their research, they demonstrated that water extracts from lyophilized fruiting bodies of this fungus, cultivated in tropical conditions, showed the greatest stimulation of neurite growth in in vitro cultures. Hazekawa et al. [33] also showed that a 14-day treatment with dried, water-dissolved *H. erinaceus* fruiting bodies exhibited neuroprotective effects in mice with MCA occlusion-induced brain ischemia. Moreover, treatment with *H. erinaceus* at the same concentration in healthy mice also showed increased NGF levels in the examined brain regions, which confirms the results of previous studies.

In 2014, Kim et al. [136] conducted in vivo and in vitro experiments combining extracts from *H. erinaceus* and *Allium sativum*. In vitro studies were performed on PC12 cells, and it was shown that the HGE extract (*H. erinaceus* mycelium enriched with garlic extract) partially delayed the apoptosis of nerve cells, leading to an increase in neurite numbers and accelerating the growth of nerve cells. HGE was found to downregulate the expression of the protein p21, which plays a significant role in the cell cycle and can stimulate nerve cell growth. Overexpression of this protein in ischemic tissues leads to cell cycle arrest and cell death. These results were confirmed in in vivo studies conducted on Mongolian gerbils (*Meriones unguiculatus*). A comparison of changes in the CA1 region of the hippocampus in animals with induced forebrain ischemia before and after HGE administration showed that the extract increased cell survival by 60%. Additionally, increased endothelial cell proliferation and vessel numbers were observed. These findings indicate the neuroprotective effect of this extract and suggest that it may aid in recovery after ischemic brain damage (Table 2).

In a study conducted by Wong et al. [50,137], the impact of the aqueous extract of fresh *H. erinaceus* fruiting bodies on sciatic nerve regeneration following crush injury in adult female Sprague-Dawley rats was investigated. It was found that the free radicals generated after the injury played a dominant role in delaying functional regeneration. Better regenerative effects were achieved with therapies aimed at counteracting the damage resulting from ischemia and reperfusion, such as antioxidants, lipid peroxidation inhibitors, and anti-inflammatory drugs. Therefore, the use of medicinal mushrooms may be a potential alternative to neurotrophic factors in peripheral nerve repair processes. Although the effectiveness of mushrooms is lower than that of neurotrophins, the aqueous extract of fresh *H. erinaceus* fruiting bodies can be used as an adjunct to neurotrophin therapy to enhance axonal regeneration in the nervous system and reduce the dosage of neurotrophins to limit potential toxic effects. The study showed that daily oral administration of the aqueous extract of fresh *H. erinaceus* fruiting bodies accelerated the regeneration of damaged peripheral nerves in rats. Additionally, scientists pointed out that *H. erinaceus* could influence neuronal functions by regulating the activity of various signaling pathways, such as the activation of cAMP response element-binding (CREB). CREB signaling plays a significant role in hippocampal long-term potentiation, which is important for learning and memory processes, as well as in CREB hyperphosphorylation (Table 2).

Research conducted by Üstün and Ayhan [56] demonstrated that the aqueous extract of fresh *H. erinaceus* fruiting bodies (HE) has the ability to prevent neuronal death and promote axonal regeneration in an experimental axonal injury model. In that study, the effects of HE were compared with NGF. Both HE and NGF showed neuroprotective and regenerative effects on damaged peripheral sensory neurons, but the protective efficacy of HE treatment or the combination of HE with NGF showed higher protective activity. It was observed that the regenerative ability of the NGF + HE combination was stronger than that of the individual substances alone. Therefore, HE or its combination with NGF may represent an effective and safe therapeutic option for the treatment of peripheral nerve injuries.

Scientists suggest that NGF and HE may act through different protective and regenerative mechanisms while also exhibiting antioxidant and anti-inflammatory properties. It is worth noting that the neuroprotective activity of HE is higher than that of NGF, possibly due to the presence of additional antioxidant and anti-inflammatory properties. The results obtained indicate that HE may become a promising candidate for preventing neuronal death, improving nerve function, and treating peripheral nerve injuries. Zhang et al. [60] also conducted research on the impact of water extracts from *H. erinaceus*. The study showed that HE exhibits protective properties against L-Glu-induced neurotoxicity in DPC12 cells, mainly through the regulation of mitochondrial pathways. Additionally, experiments conducted on mice with Alzheimer’s disease induced by AlCl3 and D-gal confirmed the protective action of HE, which may also affect neurotransmitter modulation. Furthermore, a significant increase in the concentration of acetylcholine (ACh) and choline acetyltransferase (ChAT) activity was observed in the serum and hypothalamus of mice with Alzheimer’s disease (AD) after HE administration. Patients with AD show reduced ChAT activity and insufficient ACh content in the brain, leading to impaired learning and memory abilities. Based on these results, it can be inferred that HE may be a potential candidate as a neuroprotective substance in the treatment and prevention of neurodegenerative diseases (Table 2).

Travato et al. [61] also conducted research that demonstrated the neuroprotective properties of *H. erinaceus*. During three months of supplementing HE in rats, increased expression of the genes responsible for cell protection, especially HSP70, HO-1, and TRX, was observed, leading to increased synthesis of lipoxin A4 (LXA4) in various regions of the rat brain. The largest inductions of LXA4 were observed in the cerebral cortex, hippocampus, striatum, and cerebellum. This discovery is of significant importance for the pathogenesis of Alzheimer’s disease (AD) and Parkinson’s disease (PD), especially in the context of theories connecting aging, neuronal degeneration, and oxidative damage. The results also indicate that HE supplementation may modulate the nutritional regulations of key proteins responsible for brain tolerance to stress. There is a probability that HE supplementation increases the redox potential, leading to the induction of genes encoding protective proteins. This may strengthen neurons sensitive to stress and protect them from apoptosis-induced neurodegeneration. These results are consistent with the study conducted by Hsu et al. [125]. The scientists conducted research using the MPTP mouse model, which showed that HEM may increase dopamine levels in patients with Parkinson’s disease (PD).

Jang et al. [124] conducted research to assess the impact of *H. erinaceus* supplementation (HE) on neuroprotection in pilocarpine-induced status epilepticus (SE) and to investigate the underlying mechanisms, with a focus on potential applications in the treatment of temporal lobe epilepsy (TLE). In patients with epilepsy, extensive neuronal damage, inflammation, and abnormal neurogenesis are considered contributing factors to chronic seizure occurrences. Therefore, numerous studies are being conducted to identify potential dietary supplements and functional foods that could be used in TLE treatment. Researchers found that administering doses of 60 and 120 mg/kg HE resulted in reduced neuronal death in the hippocampus 7 days after pilocarpine-induced seizures. However, high doses of HE did not show neuroprotective effects, suggesting the existence of an optimal dose range of HE that provides protection against seizure activity. Under the influence of HE treatment, reduced cyclooxygenase-2 (COX2) expression, a pro-inflammatory factor, was observed with doses of 60 and 120 mg/kg HE. These findings indicate that HE supplementation may contribute to neuroprotection in cases of SE and represent a novel candidate as a nutritional substance for treating TLE. These results confirm the previously mentioned studies cited above (Table 2).

It has been demonstrated that compounds known as 3-hydroxyhericenone F [85] and dilinoleoyl phosphatidylethanolamine [38] that are purified from the extract of dried *H. erinaceus* fruiting bodies have the ability to reduce cell death induced by endoplasmic reticulum stress. Similar properties were observed for hericenone C and regioisomers of hericenones B-D [107]. This may contribute to reducing the risk associated with cell death induced by neurodegenerative diseases (Table 1).

### 4.2. Cellular Aging Inhibition

Noh et al. [117] evaluated the inhibitory effects of six compounds isolated from *H. erinaceus* on cell aging induced by adriamycin in human skin fibroblasts (HDF) and human umbilical vein endothelial cells (HUVEC). One of these compounds, ergosterol peroxide, showed decreased senescence-associated β-galactosidase (SA-β-gal) activity, which was elevated in HUVEC cells treated with adriamycin. This suggests that one or more pure compounds may have potential in the treatment or prevention of age-related diseases in humans (Table 2).

### 4.3. Improvement of Cognitive Function

Adult hippocampal neurogenesis is one of the most interesting aspects of neurogenic zones in the adult brain due to its role in higher cognitive functions, especially in memory processes and some affective behaviors. In particular, neurogenesis in the dentate gyrus of the hippocampus leads to the generation of new granule cells in the adult brain and significantly contributes to lifelong plasticity. Newly formed neurons in the dentate gyrus have also been shown to be essential for mediating the beneficial effects of antidepressant treatment [138].

The first in vivo and in vitro studies conducted on wild-type mice by Brandalise et al. [126] demonstrated that oral supplementation of *H. erinaceus* significantly improved recognition memory in a behavioral test and increased spontaneous and induced synaptic activity in mossy fiber-CA3 synapses in mouse hippocampal sections. This suggests that *H. erinaceus* exerts a reinforcing effect on neuronal functions, even under non-pathological conditions.

Ratto et al. [31] conducted a study on the same animal model using aging mice. To best generalize the results and translate them to humans, researchers decided to use extracts from mycelium and fruiting bodies that mimic the supplements used by humans. It was confirmed that He1 supplementation is able to improve cognitive abilities in older mice and reverse cognitive decline in mice with signs of weakening. A restored proliferation of granule cells in the dentate gyrus and pyramidal neurons in the CA3 layer of the hippocampus was observed. Additionally, the presence of progenitor cells in the granule cells of the DG region was observed, supporting the hypothesis that the *H. erinaceus* extract promoted neurogenesis in the hippocampus of adult mice. These results are consistent with a study conducted by Ryu et al. [86], where it was demonstrated that a 28-day administration of an ethanol extract of HE significantly increases the proliferation and survival of precursor cells in the hippocampus without affecting the proportions of differentiated neurons. Scientists suggest that the molecular mechanism underlying this phenomenon may be related to the production of NGF, which regulates the proliferation and differentiation of neural stem cells. The findings of Ratto et al. [31] are also interesting, as based on observations of increased cell proliferation in the cerebellum, they suggest the presence of newly formed, immature neurons in the cerebellar cortex of mice treated with *H. erinaceus*, despite the cerebellum being considered a “non-neurogenic” area.

Mori et al. [21] conducted a double-blind trial on Japanese men and women with diagnosed mild cognitive impairment. The study aimed to investigate the effectiveness of oral administration of *H. erinaceus* in improving cognitive functioning, measured with the Revised Hasegawa Dementia Scale (HDS-R). Cognitive function scores increased with a longer duration of administration. Laboratory research showed no adverse effects of *H. erinaceus*, and the substance proved effective in improving mild cognitive impairment.

Mori et al. [34] also conducted a study to investigate the impact of oral administration of *H. erinaceus* fruiting body powder for 23 days in mice with cognitive and learning deficits induced by Aβ(25-35) peptide administration. Researchers assessed learning function and demonstrated that *H. erinaceus* supplementation improved cognitive deficits induced by the amyloid peptide. These results suggest a promising effect of this supplement in treating cognitive function disorders. These findings are consistent with the results of a pilot study conducted by Vigna et al. [37] on 77 overweight patients (62 women, 15 men), which found that *H. erinaceus* supplementation contributed to improving depression-anxiety mood disorders and the quality of nocturnal rest. These effects were still present eight weeks after the end of *H. erinaceus* supplementation, suggesting that it may affect neuronal plasticity. Improvement in mood disorders was associated with changes in pro-BDNF levels and the pro-BDNF/BDNF ratio in peripheral blood.

Similar results were obtained by Lee et al. [53], who conducted a study on the long-term intake of EAHEM and demonstrated improved learning ability by improving memory behavior. Similar results were also obtained by Tzeng et al. [133], who conducted a study on transgenic APP/PS1 mice. Chiu et al. [26] observed that *H. erinaceus* supplementation led to an increase in the levels of neurotransmitters such as dopamine, serotonin, and norepinephrine in the hippocampus of mice deprived of mobility. These results suggest the antidepressant effects of *H. erinaceus*, which is consistent with the findings of Nagano et al. [130], who showed that *H. erinaceus* supplementation led to a reduction in depression and anxiety in 30 women after 4 weeks of supplementation. Saitsu et al. [128] conducted a comparative study in which the consumption of cookies containing *H. erinaceus* for 12 weeks showed alleviation of symptoms of short-term memory impairment and improved cognitive function in 31 participants.

Li et al. [51] confirmed the effectiveness of *H. erinaceus* in alleviating neurodegenerative disorders in patients with mild Alzheimer’s disease. Meanwhile, Rodriguez and Lippi [127] conducted a study on the rTg4510 mouse model, which exhibits tau protein pathology and serves as a model for Alzheimer’s disease. Researchers did not confirm an improvement in cognitive function following *H. erinaceus* supplementation but did observe an anxiolytic effect. Rossi et al. [57] demonstrated a positive impact of *H. erinaceus* on spatial memory in mice (Table 2)

### 4.4. Anti-Neuroinflammatory and Antioxidant Effects

The occurrence of neurodegenerative diseases is closely related to neuroinflammation. Studies have shown that microglial cell activation can be induced by lipopolysaccharide (LPS), leading to the generation of significant amounts of ROS, nitric oxide (NO), and pro-inflammatory cytokines such as interleukin-6 (IL-6) and tumor necrosis factor-alpha (TNF-α). These factors are responsible for neuronal damage and ultimately contribute to the development of neurodegenerative diseases. Brain neurons and endothelial cells are responsible for the production of NO through the transformation of arginine involving the NO synthase enzyme, which is activated by calcium ions (Ca^2+^). This process also requires the involvement of NADPH and O_2_.

There are three isoforms of NO synthase: endothelial NO synthase (eNOS), neuronal NO synthase (nNOS), and inducible NO synthase (iNOS). Higher levels of iNOS can be observed in many cell types, such as glial cells, macrophages, skeletal muscles, neurons, platelets, and leukocytes. iNOS expression is usually elevated in response to inflammatory conditions and oxidative stress [53,139]. iNOS activation in cells can be stimulated by LPS, TNF-α, and IL-1 [42]. It has been shown that hericenone C may inhibit iNOS expression, leading to the inhibition of LPS-induced NO production [42]. These results are consistent with the findings of Lee et al. [53], who demonstrated that EAHEM administration to mice resulted in reduced iNOS expression in the brain. These results suggest that the protective action of EAHEM may be a result of reduced oxidative stress and inflammatory state, which is consistent with a previous study by Lee et al. [20], conducted in vivo, which showed that EAHEM reduced the levels of pro-inflammatory cytokines, including IL-1β, IL-6, and TNF-α. Additionally, reduced iNOS expression and proteins containing nitrotyrosine were observed, as well as inhibition of the p38 MAPK and CHOP signaling pathways. These results suggest that the iNOS/p38 MAPK signaling pathway may be involved in neuron survival, which can be mediated by erinacine A after brain ischemic injury.

NO also acts as a mediator in the nervous system, influencing learning and memory. The action of reducing IL-6 and TNF-α levels was also confirmed by Chiu et al. in 2018 [26]. Moreover, researchers demonstrated reduced NF-κB and IκB protein expression in the cytosolic fraction of the hippocampus in restraint-stressed (RS) animal models, which was normalized in mice supplemented with HE. NF-κB is a key transcription factor that translocates to the cell nucleus and activates the transcription of many important genes, such as pro-inflammatory cytokines and induced enzymes like inducible nitric oxide synthase (iNOS) and cyclooxygenase (COX)-2. Therefore, targeting the NF-κB pathway may be an interesting therapeutic strategy for the treatment of depression, as inflammatory states play a significant role in the development of this disorder. Reduced NF-κB protein expression and phosphorylation of IκBα (p-IκBα) were also confirmed by Wang et al. in 2019 [42]. In their study, they demonstrated that hericenone C can inhibit Kelch-like ECH-associated protein 1 (Keap1) while increasing nuclear factor erythroid 2-related factor 2 (Nrf2) transcription factor activity and heme oxygenase-1 (HO-1) expression. Based on this data, it can be inferred that the mechanism of action of hericenone C involves inhibiting the expression of IκB, p-IκBα, and iNOS while activating the Nrf2/HO-1 pathway.

Tsai et al. [54] conducted an experiment on 15-month-old mice fed a high-fat and high-sucrose diet (HFSD), also showing that both HEM and EA act as anti-inflammatory agents by reducing mRNA expression of TNF-α and IL-1β in the mouse hippocampus. Mice treated with HEM showed increased mRNA expression of NGF and NeuN. It was also shown that EA and HEM can reverse spatial learning deficits. They also demonstrated that both HEM and EA effected reductions in body weight, abdominal fat tissue, blood glucose levels, total serum cholesterol, and liver triglycerides. Based on these results, it can be concluded that HEM may be a potential health-promoting supplement that minimizes aging progression and neurodegeneration induced by obesity by reducing metabolic disorders and neuroinflammatory cytokines, as well as by increasing neurogenesis factors.

The antioxidant activity of *H. erinaceus* has also been identified as another significant aspect alongside its anti-inflammatory activity.

Mainly, the antioxidant activity of *H*. *erinaceus* is due to the presence of polysaccharides (β-glucans) and diterpenoids (hericenones, erinacines) [76]. However, several researchers have stated that there is a close link between the antioxidant activity of *H. erinaceus* and the presence of flavonoids and polyphenols [140,141].

Oxidative stress is a significant component in the pathogenesis of neurodegenerative diseases, including Alzheimer’s and Parkinson’s diseases. At the core of these molecular mechanisms lies the accumulation of ROS and nitrogen species (RNS), resulting in damage to lipids, proteins, and organelles, disruption of mitochondrial membranes, and apoptosis, ultimately leading to neuronal death [115,142]. A growing body of evidence suggests that inflammatory reactions and the release of pro-inflammatory cytokines also lead to oxidative stress and disrupt redox homeostasis associated with mitochondrial dysfunction [139].

Research has demonstrated that the administration of hot water extracts from HE leads to improved scavenging of free radicals and inhibition of lipid peroxidation [143]. Polysaccharide extracts from *H. erinaceus* have also been found to reduce peroxidation levels, increase antioxidant enzyme activity, and enhance free radical scavenging activity [125,136,144]. Previous studies conducted on an MPTP-induced mouse model showed that administration of EAHEM counteracted oxidative stress. It was observed that EAHEM administration led to a decrease in nitrotyrosine and 4-HNE expression while restoring motor function in the rotarod test in mice. These findings suggest that *H. erinaceus* may exhibit protective potential against brain oxidative damage through its antioxidant action. Protection against oxidative stress may play a significant role in preventing and alleviating neurodegenerative diseases.

These findings were corroborated by Amara et al. [121], who investigated the in vitro neuroprotective role of HE biomass preparation against DEHP-induced neurotoxicity. The scientists demonstrated that pretreatment with HE significantly attenuated cell death induced by DEHP. This protective effect may be attributed to HEs ability to reduce intracellular reactive oxygen species levels, preserve the activity of respiratory complexes, and stabilize the mitochondrial membrane potential. Kushairi et al. 2019 [115] also evaluated the protective and anti-inflammatory effects of aqueous (HE-HWA) and ethanol (HE-ETH) extracts from *H*. *erinaceus* fruiting bodies. In their in vitro studies, the scientists found that HE-ETH exhibited more potent neuroprotection and more effective anti-inflammatory activity than HE-HWA, inhibiting NO production in HT22 mouse hippocampal cells and LPS-activated BV2 microglia. The mechanisms of neuroprotection included enhancing antioxidant activity, mitochondrial function, and anti-apoptotic effects.

Studies suggest that treatment with erinacine A acts by protecting against endoplasmic reticulum (ER) stress, which is associated with increased neurotoxicity and neuronal apoptosis. The main mechanism of action involves the activation of the RE1α/TRAF2, JNK1/2, and p38 MAPK pathways, leading to the regulation of various factors such as CHOP, IKB-β, NF-κB, Fas, and Bax. These factors can influence the process of apoptosis and protect neuronal cells. Activation of the RE1α/TRAF2 pathway is linked to the regulation of the ER stress response and may play a role in protecting neurons from oxidative stress and other factors. The JNK1/2 and p38 MAPK pathways are also involved in the ER stress response and the regulation of apoptotic and cell survival processes in neuronal cells. CHOP expression may impact the process of apoptosis, while IKB-β and NF-κB may be involved in regulating inflammation. Fas and Bax expression can affect the course of apoptosis by activating appropriate pathways. In summary, erinacine A acts on various signaling pathways that may play a crucial role in protecting neuronal cells from stressful factors such as ER stress. The protective action of erinacine A may contribute to reducing neurotoxicity and apoptosis, which could be significant in the prevention and treatment of neurodegenerative diseases (Table 2).

## 5. Toxicology Studies

Many of the studies mentioned above have confirmed the significant therapeutic properties of *H*. *erinaceus*. However, it is necessary to consider its safety and the associated risks of its use. Experimental analyses based on the action of *H*. *erinaceus* mycelium components suggest that they are potentially safe and do not induce side effects. In the study by Lai et al. (2013) [2], no cytotoxic effects were observed with aqueous solutions of *H*. *erinaceus* on NG108-15 cells and human lung fibroblasts. This is consistent with the results of Lew et al. (2020) [118], who also did not observe such effects in studies conducted on PC-12 cells. Additionally, in the research conducted by Li et al. (2018) [96], it was shown that all rats treated with EAHE mycelia (at a dose of 5 g/kg body weight) did not exhibit any signs of treatment-related toxicity or mortality. This suggests the low toxicity of EAHE mycelia in the context of their effect on animal growth. Additional studies have shown that EAHE did not cause developmental defects in these animals, even at doses of up to 2625 mg/kg. Previous studies in rats also demonstrated that oral administration of EAHE at doses of up to 3 g/kg for 28 days did not induce any signs of toxicity, morbidity, or mortality in rats of both sexes [145]. A subchronic toxicity study was also conducted in rats using water extracts of *H*. *erinaceus* at doses up to 1000 mg/kg body weight for 90 days. No toxicity, morbidity, or mortality signs were observed [146]. Chen et al. (2022) also conducted subchronic toxicity and genotoxicity studies of *H*. *erinaceus* β-glucan extract preparation [147]. The study was conducted in Sprague-Dawley rats, and based on the results, the No Observed Adverse Effect Level (NOAEL) for the β-glucan extract preparation from *H*. *erinaceus* was determined to be 2000 mg/kg body weight/day, which was the highest tested dose. These results suggest that it is unlikely for the β-glucan extract from *H*. *erinaceus* to induce any genotoxic effects. These findings are consistent with studies by Li et al. (2014) [148], which showed that *H*. *erinaceus* mycelia were not mutagenic in the bacterial reverse mutation test (Ames test), in vitro chromosomal aberration test, and in vivo micronucleus test on erythrocytes, both with and without metabolic activation.

## 6. Conclusions

Research on *H. erinaceus* and its neuroprotective properties has shown promising results and provides an excellent starting point for further studies to gain a more in-depth understanding of this species and prepare potential drugs/dietary supplements. Fungi show immense potential as polypharmaceutical drugs due to their rich and complex chemistry and diverse forms of bioactivity. They contain many chemical compounds, such as polysaccharides, triterpenes, alkaloids, flavonoids, and other components that exhibit potential therapeutic effects. However, standardization of dietary supplements based on medicinal mushrooms is still in its early stages of development. There are no uniform standards and protocols regarding the quality and composition of mushroom supplements. Difficulties in standardization and isolation of specific substances arise from the fact that secondary metabolites, widely analyzed in *H. erinaceus*, are often produced in response to various stress conditions. This makes it challenging to ensure consumers receive products of appropriate quality with expected health benefits. To guarantee the quality and effectiveness of supplements with medicinal mushrooms, further scientific research is necessary, along with the development of appropriate standards and protocols for the production, extraction, standardization, and quality control of these products. It is also essential to monitor the chemical composition and biological activity of fungi and develop suitable tests and methodologies to evaluate the effectiveness and safety of dietary supplements with mushrooms. All these efforts will contribute to the development of more advanced and personalized therapies based on medicinal mushrooms and enable consumers to use high-quality products with potential health benefits.

Further research and standardization work on dietary supplements based on *H. erinaceus* is essential to ensuring their effectiveness and safety in the context of neuroprotection in humans. Increased availability of pure analytical standards and the development of techniques for isolating and characterizing active ingredients will be crucial in the standardization and production of high-quality dietary supplements based on *H. erinaceus*. Furthermore, to assess the effectiveness and safety of using mushrooms for neuroprotection in humans, further research, including clinical trials, is necessary. Clinical trials will evaluate the impact of mushrooms on cognitive functions, memory, and other neuroprotective indicators in patients with neurodegenerative diseases. Understanding the mechanisms of action of active mushroom ingredients at the molecular and cellular levels may lead to the further development of mushroom-based therapies and the identification of specific compounds with potential neuroprotective effects.

## Figures and Tables

**Table 1:** Biological activity of secondary metabolites isolated from *Hericium erinaceus*.

Components	Biological Activities	Tests	Test Cell Lines/Organisms	Reference
Erinacine A	induced 250.1 ± 36.2 pg/mL NGF synthesis *;	In vitro	mouse astroglial cells	[40]
	enhanced neurotrophin production (increased NGF mRNA)		1321N1 human astrocytoma cells	[41]
Erinacine B	induced 129.7 ± 6.5 pg/mL NGF synthesis *;	In vitro	mouse astroglial cells	[40]
	enhanced neurotrophin production (increased NGF mRNA)		1321N1 human astrocytoma cells	[41]
Erinacine C	induced 299.1 ± 59.6 pg/mL NGF synthesis *;	In vitro	mouse astroglial cells	[40]
	enhanced neurotrophin production (increased NGF mRNA, increased BDNF mRNA);		1321N1 human astrocytoma cells	[41]
	preventing neuroinflammation (reduced NO, IL-6, and TNF-α, inhibiting the expression of NF-κB and phosphorylation of IκBα)		BV2 microglial cells	[42]
Erinacine D	induced 141.5 ± 18.2 pg/mL NGF synthesis *	In vitro	rat astroglial cells	[43]
Erinacine E	induced 105 ± 5.2 pg/mL NGF synthesis *;	In vitro	rat astroglial cells	[44]
	enhanced neurotrophin production		1321N1 human astrocytoma cells	[41]
Erinacine F	induced 175 ± 52 pg/mL NGF synthesis *	In vitro	rat astroglial cells	[44]
Erinacine H	induced 31.5 ± 1.7 pg/mL NGF synthesis *	In vitro	rat astroglial cells	[45]
Erinacine P	significant neurite outgrowth-promoting effects	In vitro	PC12 cells	[46]
Erinacine S	neurite outgrowth of primary neurons from both the CNS and PNS are significantly enhanced	In vitro	Mouse cortical neuron cultures Rat dorsal root ganglion (DRG) neuron cultures	[82]
Erinacine T	significant neurite outgrowth-promoting effects	In vitro	PC12 cells	[46]
Erinacine U				
Erinacine V				
Erinacine W (non-natural analogue)	stimulated neurite outgrowth	In vitro	PC12 cells	[47]
Erinacine X (non-natural analogue)				
Erinacine Y (non-natural analogue)				
Erinacicne ZA (non-natural analogue)				
Erinacine Z1	increased NGF mRNA	In vitro	1321N1 human astrocytoma cells	[41]
Hericenone A	cytotoxicity	In vitro	HeLa cells	[83]
Hericenone C	induced 23.5 ± 1.0 pg/mL NGF synthesis *	In vitro	mouse astroglial cells	[48]
Hericenone D	induced 10.08 ± 0.8 pg/mL NGF synthesis *			
Hericenone E	induced 13.9 ± 2.1 pg/mL NGF synthesis *;		mouse astroglial cells	[48]
	NGF-induced neurite outgrowth		PC12 cells	[35]
Hericenone F	reduced NO generation-anti-inflammatory effect	In vitro	RAW264.7 cells	[49]
Hericenone H	induced 45.1 ± 1.1 pg/mL NGF synthesis *	In vitro	mouse astroglial cells	[84]
3-Hydroxyhericenone F	protective activity against endoplasmic reticulum (ER) stress	In vitro	culture medium of Neuro2a cells	[85]
Hericerin Isohericerinol A	increase of the NGF level in a dose-dependent manner;	In vitro	C6 glioma cells	[86]
Isohericerinol A Corallocin A	increased the expression of BDNF protein			
Erinacene D	inhibited the induction of iNOS and ICAM-1 mRNA; suppress TNFα–induced NF-κB transcriptional activity	In vitro	HaCaT cells	[87]
3,4-Dihydro-5-methoxy-2-methyl-2-(40-methyl-20-oxo-30-pentyl)-9(7H)-oxo-2h-furo[3,4-h]benzopyran	exhibited high neurite outgrowth-promoting activity	In vitro	PC12 cells	[39]
DLPE (dilinoleoyl-phosphatidylethanolamine)	protection against neuronal cell death caused by endoplasmic reticulum (ER) stress and oxidative stress	In vitro	Neuro2a cells	[38]

* The amounts of NGF secreted into the culture medium.

**Table 2:** The impact of various extracts from *Hericium erinaceus* on studied cell lines or organisms.

**Tests**	**Type of Extract, Dose and Dosage**	**Biological Activities**	**Test Cell Lines/Organisms**	**References**
In vitro	Ethanolic extract, *H*. *erinaceus* fruiting bodies	promotion of NGF mRNA expression in a concentration-dependent manner through activation of the JNK pathway	1321N1 human astrocytoma cells	[36]
	Ethanolic extract, *H*. *erinaceus* mycelia	inhibited the cell cycle G1 distribution as a result of the generation of the ROS and mTOR/p70S6K/NF-κB/p21 pathway	human colon cancer cell line DLD-1 (CCL-221) and human colorectal carcinoma cell line HCT-116 (CCL-247)	[113]
	Ethanolic extract, *H. erinaceus* mycelia, and a solution of erinacine A	modulate multiple signalling pathways involved in neuronal survival and cell death pathways	Neuro-2a cells	[23]
	Ethanolic extract, *H. erinaceus* mycelia enriched erinacine A	exerted an anti-apoptotic function by modulating the transcription factors p53 and NF-κB and their downstream events in cell lines,	neuroblastoma K-N-SHMJD78 cells	[114]
		enhancing neurite growth of primary cortical neurons in a dose-dependent manner	primary cultures of neonatal rat cortical neuronal cells	[96]
	Ethanolic and hot water extract, *H. erinaceus* mycelia	exerted potent neuroprotection and NO-suppressing anti-inflammatory activity	HT22 cells	[115]
	Ethanolic extract from *H*. *erinaceus*	promoted the normal cultivation of the nerve and glial cells; influence on the process of myelination	cultured of WISTAR rat cerebellum cells	[116]
	Methanolic extract, *H*. *erinaceus* fruiting bodies	inhibitory effects against cellular senescence in human primary cells,	Human dermal fibroblasts (HDFs), human umbilical vein endothelial cells (HUVECs), endothelial cell growth medium-2(EGM-2)	[117]
	Aqueous extract, *H. erinaceus* fruiting bodies	induced 45.67 ± 0.79 pg/mL NGF synthesis * increased neurite extension	NG108-15 cells	[2,55]
		the protective abilities of *H*. *erinaceus* treatment and its combination with NGF were significantly higher than NGF treatment alone	mouse PNI model	[56]
	Aqueous extract of Malaysian-grown *H*. *erinaceus*	increase neurite extension; protective effect against oxidative stress	NG108-15 cells	[2]
		neuroprotective effects against high-dose corticosterone-induced oxidative stress	PC12 cells	[118]
	Aqueous extract, *H*. *erinaceus* mycelia and fruiting body, garlic extract	have the synergistic effect of the mycelium and garlic extracts on neuroprotective activity	PC12 cells	[119]
	Aqueous extract, *H*. *erinaceus* mycelium	neuroprotective effect	an L-Glu-induced DPC12 cellular apoptosis model	[60]
	AuNPs using the hot aqueous extract of *H*. *erinaceus* fruiting bodies	have potential neuronal differentiation and stimulated neurite outgrowth	PC12 cells	[120]
	*H*. *erinaceus* biomass, a powder containing mycelium and primordia	increased PC12 cell survival against DEHP insult; induces anti-apoptotic activity; reduces intracellular reactive oxygen species levels	PC12 cultured in DMEM	[121]
	Enzymatic hydrolysates from *H*. *erinaceus*	more effective antioxidative and superoxide radical scavenging activity (compared to water and organic solvent extracts); neuroprotective effects against H_2_O_2_	PC12 cells	[122]
	Biopolymer from the liquid culture broth of *H. erinaceus* mycelium	enhanced the growth of rat adrenal nerve cells; both nerve growth factors also improved the growth of PC12 cells	PC12 cells	[58]
	Polysaccharide extracts from fruiting bodies *H*. *erinaceus*	antioxidant and neuroprotective effects on Aβ-induced neurotoxicity in neurons	PC12 cells	[59]
In vivo	A solution of erinacine A (8 mg/kg body weight) dissolved in 5% ethanol and saline phosphate buffer, IGAS	Enhanced NGF and catecholamine secretion in the LC and hippocampus, and a decrease in the cerebral cortex	normal Wistar rats	[123]
	Ethanolic extract, *H. erinaceus* mycelia (50, 300, and 1000 mg/kg body weight), PO, and a solution of erinacine A (1, 5, and 10 mg/kg), IP for 5 days	reduced infarcted volume in the cortex and subcortex; reduced levels of proinflammatory cytokines such as iNOS, IL-1β, IL-6, and TNF-α in the serum	ischemic stroke in Sprague-Dawley rat	[20]
	A solution of erinacine A (1, 2, and 5 mg/day) for 5 days, IP	decreased the growth of the xenografts of CRC cells in nude mice by inhibiting cell proliferation and promoting apoptosis	BALB/c-nu mice	[113]
	Ethanolic extract, *H. erinaceus* mycelia (10.76 mg and 21.52 mg), PO, and a solution of erinacine A (1 mg/kg body weight) IP for 5 days	the signalling molecules affected by erinacine A included the survival factors PAK1, cdc42, AKT, LIMK2, ERK, and Cofilin, IRE1á, TRAF2, ASK1, GADD45, and p21; a reduced number of apoptotic neurons	C57BL/6 mice	[23]
	Ethanolic extract, *H. erinaceus* mycelia, a solution of erinacine A and S (30 mg/kg/day) for 30 days, PO	reduced amyloid plaque burden in the cerebral cortex; increased the level of insulin-degrading enzyme (IDE) in the cerebral cortex	APPswe/PS1dE9 transgenic mice	[52]
	Ethanolic extract, *H. erinaceus* mycelia enriched erinacine A, PO	exerted an anti-apoptotic function by modulating the transcription factors p53 and NF-κB and their downstream events in Drosophila models of SCA3 disturbed by oxidative stress	Drosophila models of SCA3 (fly stocks, elav-Gal4, UAS-MJDtr-Q27, and UAS-MJDtr-Q78 flies)	[114]
	300 mg/kg/day of mycelia powder and ethanolic extract for 30 days, PO	reduced amyloid plaque burden in the area, including the cerebral cortex and hippocampus; increased NGF/proNGF ratio; and promoted hippocampal neurogenesis	5-month-old female APPswe/PS1dE9 transgenic mice	[22]
	Erinacine A- enriched *H*. *erinaceus* mycelia (108, 215, and 431 mg/kg/day) for 13 weeks, PO	lower oxidative stress significantly improved learning and memory	3-month-old male and female senescence-accelerated mice (SAMP8)	[53]
	Ethanolic extract, *H*. *erinaceus* (20 and 60 mg/kg) for 28 days, PO	increased hippocampal neurogenesis	male C57BL-6J mice	[27]
	Ethanolic extract of *H*. *erinaceus* (60, 120, and 300 mg/ kg body weight) for 21 days, PO	a reduction of COX2-expressing astrocytes; neuroprotective effect	male C57BL mice (a pilocarpine-induced SE model)	[124]
	An ethanolic mixture of lyophilized mycelium and sporophores of *H*. *erinaceus* (1 mg/supplement per day) for 2 months, PO	Increased recognition and memory performance in mice during aging; reduced cognitive decline	male C57BL-6J mice	[31]
	Methanolic extract, *H*. *erinaceus* mycelia (1 g/ kg), and a solution of erinacine A (43 mg/kg) for 18 weeks, PO	decreased neuronal loss; higher NGF biosynthesis; performed better in spatial learning; increased mRNA expression levels of TNFa and IL-1b in the hippocampus	C57BL/6 mice	[54]
	5% freeze-dried powder of fruiting bodies of *H*. *erinaceus*, PO	increased NGF mRNA in the hippocampus	male ddY mice	[36]
	Powder of *H*. *erinaceus* mycelia (100, 200, and 400 mg/kg body weight) for 4 weeks, PO	antidepressant-like effect; increased BDNF, TrκB, and PI3K expressions in the hippocampus; reduced IL-6 and TNF-α levels	restraint stress-induced depression in ICR mice	[26]
	400 mg mycelia and 100 mg dried fruiting body extract of *H*. *erinaceus* for 2 months, PO	increasing glutamatergic synaptic drive in the hippocampus; increased general locomotor activity but did not affect spatial memory	C57BL-6J mice	[57]
	The powdered fruiting bodies of *H*. *erinaceus* (5% *w*/*w*) for 23 days, PO	prevented the cognitive deficits induced by the administration of Aβ(25–35)	male 5-week-old ICR mice	[34]
	The powdered mycelia of *H*. *erinaceus* (0.1 g/kg, 0.3 g/kg, and 1 g/kg) for 30 days, PO	reduced oxidative stress; increase in dopamine levels	male C57BL/6Narl mice treated with 1-methyl-4-phenylpyridinium (MPTP)	[125]
	*H*. *erinaceus* biomass, a powder containing mycelium and primordia (200 mg/kg body weight) for 3 months, IGAS	neuroprotective effect; increased expression of genes, particularly HSP70, HO-1, and TRX), leading to an increase in LXA4 synthesis in various regions of the brain.	male Sprague-Dawley rats	[61]
	Aqueous extract, Malaysian-grown *H*. *erinaceus* fruiting bodies (10 or 20 mL kg^−1^ body weight per day) for 14 days, PO	promote the regeneration of injured rat peroneal nerves in the early stages of recovery	adult female Sprague-Dawley rats	[50]
	Aqueous extract, *H*. *erinaceus* fruiting bodies, for 14 days, PO	increased level of NGF in cortex, striatum and hippocampus	male ddY mice subjected to MCA Occlusion	[33]
	Aqueous extract, *H*. *erinaceus* mycelium (0.3, 1, and 3 g/kg body weight) for 4 weeks, PO	neuroprotective effect	AD mouse	[60]
	The supplement Micotherapy Hericium (Noceto, Parma, Italy) (contains mycelium and fruiting body extract of *Hericium erinaceus* in a ratio of 4/1), corresponding to 0.025 g/g body weight for 2 months, PO	increasing glutamatergic synaptic drive, novelty exploration behaviour, and recognition memory in the hippocampus	C57BL-6J mice	[126]
	Supplementation of *H*. *erinaceus* (Host Defense Mushrooms, Fungi Perfecti, LLC., Olympia, WA, USA) through wet food for 4 months, PO	anxiolytic effects; no improvements in spatial memory	rTg4510 tau mouse model	[127]
In vivo/ Clinical trial	Aqueous and ethanolic extract, *H*. *erinaceus* supplementation (80% mycelia and 20% fruiting body), 1.2 g per capsule; 3 capsules/day for 8 weeks; PO	decreased depression, anxiety, and sleep disorders	seventy-seven volunteers (62 females and 15 males) with a body mass index (BMI) ≥ 25 kg/m^2^	[37]
	Aqueous extract, *H*. *erinaceus* mycelium, 350 mg/capsule containing 5 mg/g erinacine A (EAHE) for 49 weeks; PO	reduced cognitive decline	patients with age > 50 years and diagnosis of probable AD	[51]
	Dried fruiting bodies of *H*. *erinaceus*, 250 mg tablets containing 96% of *H*. *erinaceus* dry powder three times a day for 16 weeks, PO	improved cognitive function	a double-blind, parallel-group, placebo-controlled trial was performed on 50- to 80-year-old Japanese men and women diagnosed with mild cognitive impairment	[21]
	0.8 g of the powdered fruiting body of *H*. *erinaceus;* 4 capsules/day for 12 weeks; PO	improved cognitive function	randomized, double-blind, placebo-controlled parallel-group	[128]
	5 g/day of the lyophilized *H*. *erinaceus* for a 6-month period; PO	improved cognitive function	fifty elderly individuals with disabilities	[129]
	Aqueous extract, 0.5 g of the powdered fruiting bodies of *H*. *erinaceus* per cookie, 4 cookies daily for 4 weeks; PO	decreased depression, anxiety	a double-blind, parallel-group, placebo-controlled trial was performed on thirty middle-aged females in menopause	[130]
	Patented extraction, Amycenone^®^, *H*. *erinaceus* fruiting body extract (0.5% hericenones and 6% amyloban), 1950 mg/tablet (Amyloban^®^ 3399) 6 tablets, divided into 2 or 3 doses /day for 6 months; PO	improved cognitive function	86-year-old male patient	[131]
	Patented extraction, Amycenone^®^, *H*. *erinaceus* fruiting body extract (0.5% hericenones and 6% amyloban), 1950 mg/tablet (Amyloban^®^ 3399) 6 tablets, divided into 2 or 3 doses /day for 4 weeks; PO	decreased depression, anxiety, and sleep disorders	8 female healthy participants	[132]

* The amounts of NGF secreted into the culture medium.

## Data Availability

Not applicable.
